# Performance of Laser-Clad Transition Layers on H13 Steel

**DOI:** 10.3390/ma18071418

**Published:** 2025-03-23

**Authors:** Junbo Zhang, Bing Du, Fuzhen Sun, Yang Liu, Yan Li

**Affiliations:** State Key Laboratory of Advanced Forming Technology and Equipment, China Academy of Machinery, Science & Technology, Beijing 100044, China

**Keywords:** H13 steel, laser cladding, transition layer, nickel-based alloys, interfacial bonding

## Abstract

This study addresses the crack formation problem when laser cladding CoCrFeNiAl high-entropy alloy onto H13 hot-work die steel, aiming to identify suitable transition layer materials. Five nickel-based alloys—Inconel 718, Inconel 625, Hastelloy X, FGH4096, and FGH4169—are selected as alternatives. Three-point bending and hot tensile tests are conducted to assess performance under different stress directions. Test results show that the FGH4096 and FGH4169 coatings fail due to insufficient element diffusion and weak interfacial bonding. Cracks appear at the coating–substrate interface of Inconel 625 and Hastelloy X. In contrast, Inconel 718 performs best, with excellent thermal expansion matching and strong stress resistance. In the three-point bending test, the specimens with Inconel 718 transition layers did not show cracks during the loading process, while specimens with some other alloy transition layers cracked or fractured, which proves that Inconel 718 can effectively enhance the bonding force between the coating and the substrate and improve the material’s performance under bending stress. In the hot tensile test, the stress–strain curve of Inconel 718 is at a high position with a high yield strength, showing excellent resistance to plastic deformation and significantly improving the performance of the nickel-based layer under hot tensile conditions. Therefore, Inconel 718 is identified as the optimal transition layer material.

## 1. Introduction

In the context of the rapid advancement of the modern manufacturing industry, optimizing the surface properties of materials has become increasingly critical for enhancing product performance, prolonging service life, and improving production efficiency [1]. As an advanced surface engineering technique, laser cladding has demonstrated significant potential in fabricating functional coatings with tailored properties on substrate surfaces. This technology has found extensive applications in various industrial sectors, particularly in mold manufacturing and mechanical engineering fields, owing to its unique advantages in surface modification and repair [2].

H13 hot-work die steel, a medium-carbon chromium-molybdenum alloy steel, is widely employed in hot-stamping and die-casting applications due to its excellent combination of toughness, thermal fatigue resistance, and wear resistance at elevated temperatures [3]. However, under severe service conditions, H13 steel encounters significant operational challenges [4]. Empirical data indicate that after approximately 100,000 thermal cycles during hot-stamping processes, the steel surface typically exhibits pronounced degradation characteristics, including thermal fatigue cracks and wear damage. This surface deterioration not only necessitates frequent die replacements, thereby increasing production costs but also significantly compromises manufacturing efficiency [5]. To address these limitations, surface engineering through laser-cladding technology has emerged as a promising solution for enhancing the performance of H13 die steel [6].

In recent years, high-entropy alloys (HEAs) and tungsten carbide (WC)-reinforced composite coatings have emerged as prominent research frontiers in surface engineering due to their exceptional mechanical and functional properties [7]. HEAs, characterized by their unique multi-principal element design paradigm, demonstrate superior high-temperature stability, wear resistance, and corrosion resistance, which can be attributed to four core effects: high-entropy effect, lattice distortion-induced solid solution strengthening, cocktail effect, and sluggish diffusion effect [8]. The selection of HEAs is primarily motivated by their compositionally complex nature, which offers enhanced flexibility in property optimization and superior adaptability to demanding service conditions compared to conventional alloy systems.

WC, as a refractory ceramic material with exceptional hardness (≥2200 HV) and high melting point (2870 °C), has been extensively incorporated into HEA-based coatings to significantly enhance their hardness and tribological performance [9]. However, the direct laser deposition of HEAs on H13 hot-work die steel substrates presents substantial challenges. The significant mismatch in thermophysical properties, particularly the coefficient of thermal expansion (CTE) between the coating and substrate, often leads to the formation of microcracks and delamination during the laser melting process. These defects severely compromise the coating–substrate interfacial integrity and overall mechanical performance, thereby limiting the practical implementation of this technology in industrial applications [10].

Our research group has previously employed laser-cladding technology to fabricate Inconel 718 transition layers on H13 steel substrates [11]. Microstructural characterization revealed that the transition layer exhibits homogeneous composition distribution and unique microstructural features, establishing a robust metallurgical bond between the substrate and functional coating. This interfacial configuration effectively alleviates stress concentration while creating a graded hardness profile across the coating system. The resulting soft-hard composite structure not only enhances the coating’s hardness and wear resistance but also significantly improves the interfacial stability between the coating and substrate. These findings demonstrate that the strategic incorporation of transition coatings in laser-cladding processes can substantially optimize the overall performance of coating systems [12]. In the extensive research on transition layer characterization, mechanical evaluation methods such as three-point bending tests and thermal tensile tests have proven to be indispensable tools for quantitatively assessing the interfacial bonding strength in multilayer coating systems. These standardized testing methodologies provide critical insights into the mechanical integrity and stress transfer characteristics at the transition layer–substrate and transition layer–working coating interfaces [13].

The three-point bending test serves as an effective methodology for simulating the bending stress conditions encountered by materials during actual service. Through comprehensive analysis of the material’s mechanical response, including load–deflection curve characteristics, crack initiation behavior, and propagation mechanisms, this test provides valuable insights into the transition layer’s performance under complex stress states [14]. Furthermore, when coupled with SEM analysis, it enables a thorough investigation of the failure mechanisms and identification of critical factors influencing transition layer performance. Despite significant advancements in laser-cladding technology for surface engineering applications, systematic investigations focusing on transition layer behavior in three-point bending scenarios remain relatively limited.

Thermal tensile testing offers a reliable approach for evaluating material performance under simulated high-temperature service conditions, particularly for components subjected to tensile stresses in elevated temperature environments [15]. Quantitative analysis of stress–strain curve evolution during testing provides crucial data for understanding the transition layer’s thermo-mechanical behavior, including its high-temperature strength and ductility characteristics [16]. When integrated with SEM analysis, this methodology facilitates a detailed examination of high-temperature failure mechanisms and the identification of key factors governing the transition layer’s elevated temperature performance [17]. Although research on high-temperature material properties continues to advance, comprehensive studies specifically addressing transition layer behavior in thermal tensile conditions remain an area requiring further exploration.

In the context of the increasing demand for surface modification of materials, the innovation of this study is the systematic conduct of a series of three-point bending tests and hot tensile tests on five nickel-based alloys (Inconel 718, Inconel 625, Hastelloy X, FGH4096, and FGH4169) as transition layer materials. This is the first comprehensive assessment of the mechanical behavior, crack initiation, and extension of these alloys under different stress conditions. The research focuses on the laser fusion cladding of CoCrFeNiAl and WC composite coatings on H13 hot-work die steel, with a special emphasis on evaluating their performance as transition layer materials by analyzing in detail their mechanical response under different stress conditions. The practical significance of this study is to provide a scientific and reasonable material selection basis for surface modification of H13 steel and related materials. It is expected that this will improve the service life of molds, reduce production costs, and enhance manufacturing efficiency, thus promoting the development of the modern manufacturing industry, especially mold manufacturing and mechanical engineering applications. In addition, this study contributes to an in-depth understanding of the properties and failure mechanisms of transition layer materials, laying the foundation for optimized material selection. The results will also promote the wider application of laser-cladding technology in surface modification of H13 steel and related materials, which will ultimately promote the development of modern manufacturing technology.

## 2. Materials and Methods

**Figure 1:**
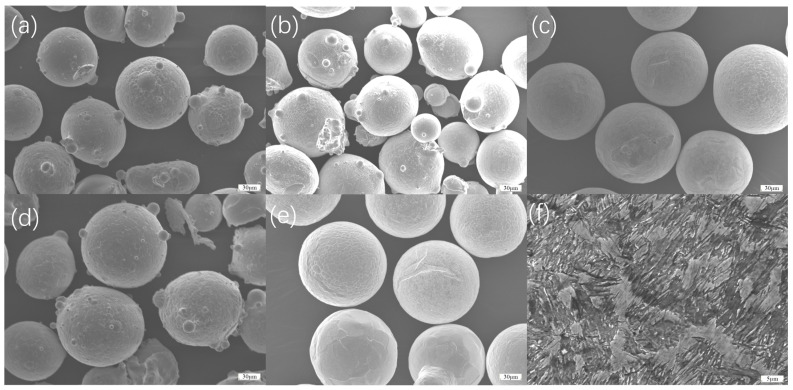
(**a**) FGH4169 powders; (**b**) FGH4096 powders; (**c**) Hastelloy X powders; (**d**) Inconel 718 powders; (**e**) Inconel 625 powders; (**f**) microstructure of H13 substrate.

H13 hot-work mold steel, which was produced by Qingdao Dite Mould Co., Ltd. in Qingdao, China, is selected as the base material.After rolling, it was quenched at 1020 °C for 30 min and tempered at 580 °C for 1 h. The microstructure of the H13 steel mainly consists of martensite, tempered carbides, and residual austenite, as shown in Figure 1f. The selected transition layer materials include FGH4169, FGH4096, Hastelloy X, Inconel 718, and Inconel 625, as shown in Figure 1a, Figure 1b, Figure 1c, Figure 1d, and Figure 1e, respectively. CoCrFeNiAl high-entropy alloy powder was selected as the working layer material. Equimolar ratio CoCrFeNiAl was used in this study, and the content of substrate and transition coating elements used are shown in Table 1. All the powder materials, including the CoCrFeNiAl high-entropy alloy powder and other powders used in the experiment, were produced by Yanbang Company in Beijing, China. Before the experiment, the high-entropy alloy powder was dried at 120 °C for 3 h, and the surface of the H13 substrate was milled, cleaned, and dried.

The LCM 6000—2000A high-power laser cladding equipment developed by the China Academy of Mechanical Engineering (Beijing, China) with an LDF 6000—100 VGP laser head system was used for cladding, and argon was used as the protective gas. Based on the experience of the research team, the process parameters were set accordingly for different materials. On the pre-treated H13 steel surface, the laser-cladding process was first carried out with a nickel-based transition coating. During the process, the processing power was set precisely at 1300 watts, the scanning speed was controlled at 7 mm/s, the overlap rate was maintained at 65%, and the powder feed rate was stabilized at 8.5 g/min to ensure that the nickel-based transition coatings could be uniformly and densely adhered to the surface of the H13 steel substrate, providing a good bonding basis and transition interface for the subsequent coatings. Subsequently, on top of the melted nickel-based transition coating, the laser melting operation of WC/CoCrFeNiAl composite coating was carried out. At this time, the processing power was increased to 1600 watts, the scanning speed was adjusted to 10 mm/s, and the powder feeding rate was set at 12.7 g/min, with the aim of utilizing the appropriate process parameters to promote the full melting, uniform spreading and good metallurgical bonding of the WC/CoCrFeNiAl composite powder with the transition coating, so as to obtain a composite coating structure with excellent performance.

**Figure 2:**
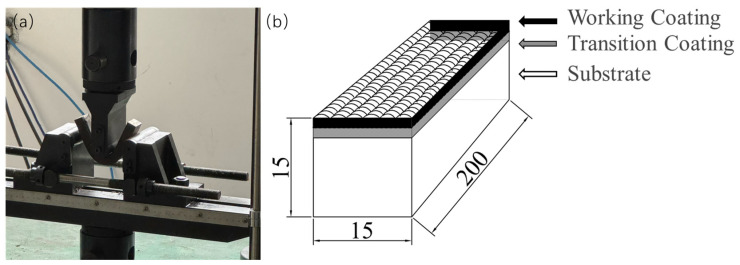
(**a**) Three-point bending process; (**b**) three-point bending specimen.

**Figure 3:**
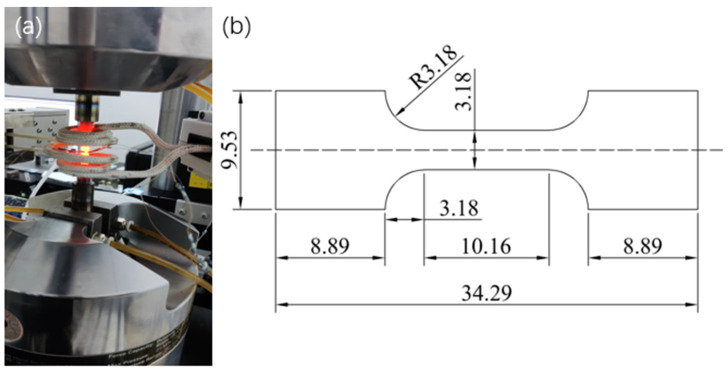
(**a**) Thermal tensile process; (**b**) thermal tensile specimen.

The three-point bending test and hot tensile test were carried out using a microcomputer-controlled universal testing machine (Model: CSS-44100) produced by Changchun Testing Machine Research Institute, China.The test parameters were set as follows: the loading rate for the three-point bending test was 0.5 mm/min; the support length of the sample was 16 mm; and the tensile loading rate for the hot tensile test was 1 mm/min. The dimensions of the equipment and samples for the three-point bending test are shown in Figure 2, and the dimensions of the equipment and samples for the hot tensile test are shown in Figure 3. Under the same test conditions, three three-point bending tests and hot tensile tests were carried out on samples with different compositions of fused cladding layers. The average of the three measurements was taken to obtain the load–deflection curve and the stress–strain curve. These tests were used to determine the first occurrence of cracks in the tensile region of the surface-reinforced layer, as well as the phenomenon of delamination fracture between the surface-reinforced layer and the substrate. The tests were also used to investigate the bond strength of different coating materials and their effect on crack extension.

## 3. Results

**Figure 4:**
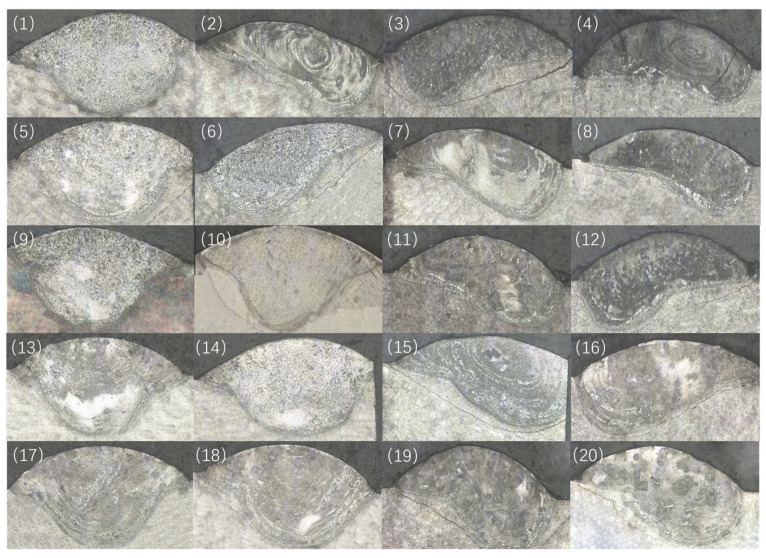
Cracks in the heat-affected zone of the cladding layer under different heat input conditions. (1) 59.52W∙mm^−2^; (2) 39.68W∙mm^−2^; (3) 29.76W∙mm^−2^; (4) 23.81W∙mm^−2^; (5) 74.40W∙mm^−2^; (6) 49.60W∙mm^−2^; (7) 37.20W∙mm^−2^; (8) 29.76W∙mm^−2^; (9) 89.29W∙mm^−2^; (10) 59.52W∙mm^−2^; (11) 44.64W∙mm^−2^; (12) 35.71W∙mm^−2^; (13) 107.17W∙mm^−2^; (14) 69.44W∙mm^−2^; (15) 52.08W∙mm^−2^; (16) 41.67W∙mm^−2^; (17) 119.05W∙mm^−2^; (18) 79.37W∙mm^−2^; (19) 59.52W∙mm^−2^; (20) 47.62W∙mm^−2^.

As shown in Figure 4, cracks appeared in the heat-affected zone when CoCrFeNiAl high-entropy alloy was directly laser cladded onto H13 steel. The specific process parameters are shown in Table 2. This is due to the significant difference in the coefficients of thermal expansion between H13 steel and CoCrFeNiAl high-entropy alloy. During the rapid thermal cycling in the laser-cladding process, the cooling phase causes the two materials to contract at different rates, resulting in the cladded layer experiencing intense tensile stress from the substrate, while the substrate is subjected to compressive stress from the cladded layer [18]. The thermal stress is concentrated in the interface region between the cladded layer and the substrate, where the microstructure and properties are relatively weak. When the stress exceeds the critical value, microcracks begin to nucleate and propagate, severely compromising the integrity of the cladded layer and reducing the bonding strength [19].

Nickel-based alloys have emerged as the preferred materials for transition coatings between CoCrFeNiAl high-entropy alloys and H13 steel substrates, owing to their optimal thermal expansion coefficient matching, excellent chemical compatibility, favorable wettability, and superior mechanical properties [20]. Specifically, Inconel 718, Inconel 625, Hastelloy X, FGH4096, and FGH4169, as representative nickel-based superalloys, demonstrate well-matched thermal expansion coefficients with both the high-entropy alloy coating and H13 steel substrate. This compatibility significantly reduces thermal stress and minimizes the risk of cracking in the heat-affected zone during laser-cladding processes [21]. Regarding chemical compatibility, these alloys exhibit remarkable stability against interfacial reactions with both CoCrFeNiAl high-entropy alloys [22] and H13 tool steels [23] at elevated processing temperatures. Furthermore, each alloy possesses distinct strengthening mechanisms (e.g., solid solution strengthening, precipitation hardening) that ensure exceptional mechanical performance under service conditions.

The elements Fe, Cr, and Ni, which co-exist in the substrate, transition coating, and working coating system and have high content, constitute a multiple diffusion system, and the quantitative characterization of the distribution of their elemental diffusion gradients by energy spectroscopy (EDS) can effectively assess the dilution rate of the interfacial region and the degree of metallurgical bonding, which provides an important basis for optimizing the design of the coating interface and the process parameters [24].

**Figure 5:**
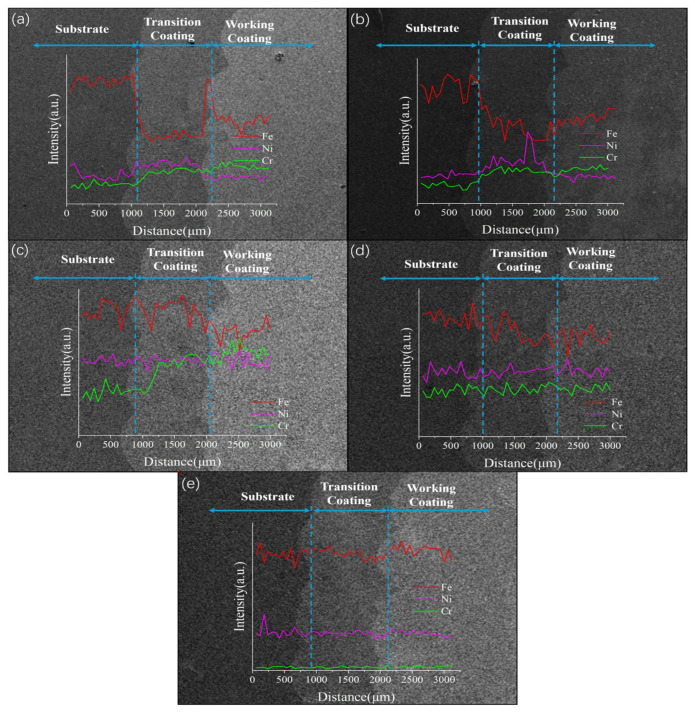
Line scans from the substrate to the working coating: (**a**) FGH4096; (**b**) FGH4169; (**c**) Hastelloy X; (**d**) Inconel 625; (**e**) Inconel 718.

As shown in Figure 5a, in GH4169, the distribution of Ni elements is not uniform, and there is obvious aggregation at the bonding place between the transition coating and the working coating, which is due to the temperature gradient at the bonding place, the difference in atomic diffusion rate, and the different physicochemical properties of different coating materials in the process of laser melting and cladding, leading to the preferential enrichment and formation of aggregation of Ni atoms in this region [25]. As shown in Figure 5b, in FGH4096, it is obvious that the iron elements in the substrate and the working coating cannot bond at the transition coating because the crystal structure and lattice constants of the transition coating do not match with those of the substrate and the working coating, and the weak inter-elemental interactions prevent the iron atoms from diffusing and bonding effectively across the interface [26].

As shown in Figure 5c, in Hastelloy X, the diffusion of iron is not uniform, which is due to the uneven distribution of the local temperature field during the fusion coating process, the existence of stress concentration areas within the coating, and the difference in the diffusion activation energy between the elements, which makes the diffusion of iron atoms in different positions at different rates; the chromium element of the transition coating is not bonded to the substrate because chromium and the substrate surface of the oxide layer, impurities, or other elements to form a stable This is because chromium and the substrate surface oxide layer, impurities or other elements to form a stable compound, preventing the formation of effective chemical bonding between chromium atoms and substrate atoms [27].

As shown in Figure 5d, Inconel 625 elements diffuse relatively uniformly due to their stable chemical composition, relatively homogeneous crystal structure, and similar diffusion coefficients and migration ability of atoms during the laser-cladding process, which allows for a more uniform distribution of elements within the coating [28]. As shown in Figure 5e, Inconel 718’s elemental diffusion uniformity is due to its good thermal stability and organizational uniformity. In the high-temperature environment generated by laser cladding, the diffusion of atoms is the more consistent driving force, and the stress distribution within the coating is uniform, which is conducive to the uniform diffusion of the elements in the entire range of coatings and the formation of stable bonding [29].

### 3.1. Three-Point Bending Test

The bond strength of five transition coatings (Inconel 718, Inconel 625, Hastelloy X, FGH4096, and FGH4169) between H13 steel and CoCrFeNiAl can be determined by a three-point bending test. This test is used to assess the first crack initiation in the tensile region of the case-hardened layer and the delamination between the case-hardened layer and the substrate [30]. The applied load corresponding to crack initiation can be easily determined from the load–deflection curve [31]. According to the basic beam theory, the cohesive strength of the bond between the transition coating and the substrate is proportional to the critical load. Therefore, this approach can be used to investigate the bond strength of different coating materials and their effect on crack propagation, and thus further investigate the role of these transition coatings on the surface toughness and durability of the material [32]. Therefore, the three-point bending test allows an in-depth analysis of their bonding properties under extreme operating conditions and the effect on the overall mechanical properties of the material.

**Figure 6:**
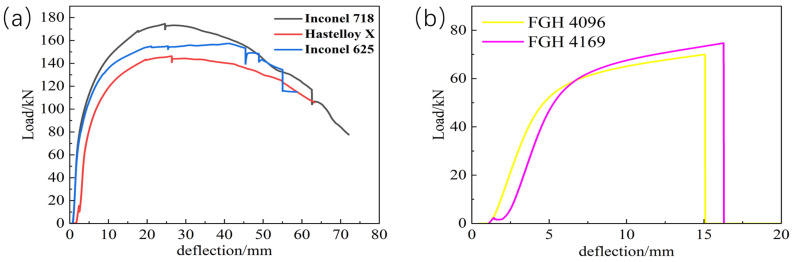
The load–deflection curve of the three-point bending specimen. (**a**) Inconel 718, Hastelloy X and Inconel 625 alloys; (**b**) FGH4096 and FGH4169 alloys.

In the three-point bending test, loads were applied to transition-coated samples of Inconel 718, Inconel 625, Hastelloy X, FGH4096, and FGH4169. As shown in Figure 6b, the FGH4096 and FGH4169 coatings were the first to experience problems as the load increased. The load–deflection curves in the three-point bending tests reflect the changes in the mechanical behavior of the materials when subjected to stresses and provide important clues for analyzing the causes of cracking of the fusion-coated layers [32]. At the initial stage of the test, as the load increases gradually, the deflection increases slowly, and the load–deflection curve usually shows a steady upward trend. This indicates that at lower stress levels, the fused cladding and the substrate can deform synergistically, and the material is in the elastic deformation stage. However, when the load reaches a certain level, due to the insufficient diffusion of certain elements, the number of chemical bonds at the interface is limited, and effective stress transfer and dissipation cannot be realized. As the load increases further, the bonding interface cannot withstand higher tensile stresses, resulting in atomic bond breakage in localized regions and microcracks begin to sprout [28]. As the load continues to increase, these cracks will gradually expand, causing the slope of the load–deflection curve to decrease. As a result, the stiffness of the material gradually decreases, eventually leading to cracking of the fusion cladding.

**Figure 7:**
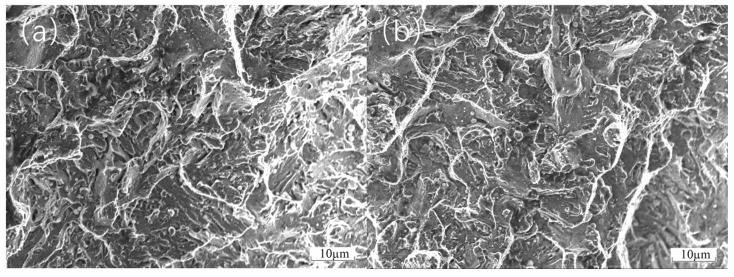
(**a**) Fracture site between FGH4169 and the substrate; (**b**) fracture site between FGH4096 and the substrate.

As shown in Figure 7, during three-point bending tests of FGH4096 and FGH4169 transition coatings, when a mixed brittle–tough fracture occurs, the fracture surfaces show complex and recognizable features under the electron microscope. In the localized area of the fracture, the characteristics of brittle fracture can be observed with a relatively smooth fracture surface and clear grain boundaries. This is due to rapid crack initiation at certain high-stress concentration points and subsequent crack extension along grain boundaries or disintegration surfaces, leaving these regions without significant plastic deformation. However, the adjacent regions show signs of ductile fracture with the presence of some tough nests. These tough nests were formed during plastic deformation due to nucleation, growth, and polymerization of micropores, which suggests that the material in these regions experienced a considerable degree of plastic flow with more active dislocation motion prior to fracture. In addition, some tear ribs can be observed in the transition region from brittle to ductile fracture. These tear ribs are the result of plastic deformation competing with brittle cracking during crack extension. As the material undergoes some degree of plastic stretching, the cracks also expand forward under the applied stress. The combination of these two processes leads to the formation of tear ribs. Together, these features clearly reveal the microscopic nature of mixed brittle–tough fracture in three-point bending tests.

**Figure 8:**
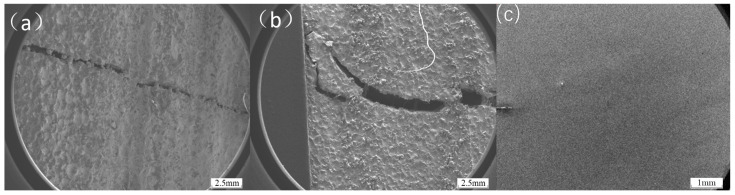
Interfacial conditions between different transition layers and the substrate after three-point bending: (**a**) Inconel 625; (**b**) Hastelloy X; (**c**) Inconel 718.

As shown in Figure 8, when observing the Inconel 625 and Hastelloy X transition coatings using a scanning electron microscope, significantly different phenomena were observed. For both Inconel 625 and Hastelloy X transition coatings, clear cracks were detected at the interface between the substrate and the working coating. This is due to the complex stress distribution generated within the cladding layer during the three-point bending process under the applied external load. Although the similar thermal expansion coefficients help reduce thermal stress effects, the mechanical stress induced by the external load cannot be ignored. During bending, different parts of the cladding layer experience varying amounts and directions of tensile and compressive stresses. When the localized stress concentration reaches the material’s yield strength, plastic deformation begins to accumulate. Once the stress exceeds the material’s fracture strength, cracks initiate. In contrast, for the Inconel 718 transition coating, no similar cracks at the interface between the substrate and the working coating were observed in the electron microscope images. This is most likely due to the excellent compatibility of Inconel 718, as its thermal expansion coefficient is better matched with both the substrate and the working coating. This results in relatively low thermal stress. Additionally, during the preparation process, good element diffusion and chemical bonding at the interface contribute to a more stable overall structure, effectively preventing crack formation even under the same observation conditions.

### 3.2. Hot Tensile Test

Laser-cladding technology has great potential in the research of material surface modification and property optimization. In this paper, an innovative coating system was constructed with H13 steel as the substrate, CoCrFeNiAl as the working coating, and Inconel 718, Inconel 625, Hastelloy X, FGH4096, and FGH4169 as the transition coatings. In order to investigate the mechanical properties and interfacial bonding characteristics of the system under thermal conditions, thermal tensile tests were conducted on the laser-melted specimens, aiming to reveal the behavior mechanism of the coatings under thermal stress and their interaction with the substrate.

**Figure 9:**
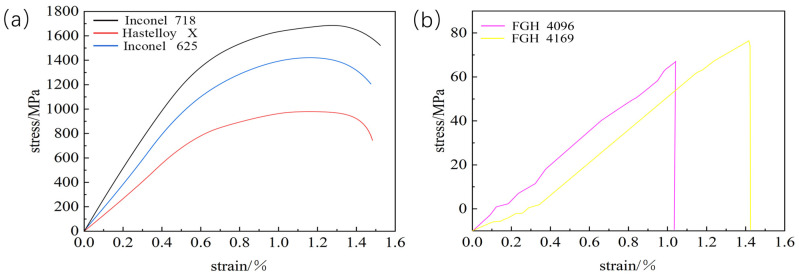
Thermal tensile stress–strain curve testing: (**a**) Inconel 718, Hastelloy X and Inconel 625 alloys; (**b**) FGH 4096 and FGH4169 alloys.

Figure 9 shows the thermal tensile curves of these five transition layer materials, which visualize the stress–strain relationship. The curve of Inconel 718 is at a high position with a remarkable stress level, indicating a high yield strength. During hot tension, it needs to withstand a large amount of stress before significant plastic deformation occurs, and it has a strong ability to resist plastic deformation in practical applications. As the strain increases, the stress first rises rapidly and then slows down, which is due to work hardening. At high temperatures, strengthening mechanisms such as dislocation motion effectively enhance its strength and deformation-resistance ability. The stress level of Inconel 625 is lower than that of Inconel 718, and its yield strength is relatively low. Under the same hot-tensile conditions, it enters the obvious plastic-deformation stage earlier. Its stress–strain curve is similar to that of Inconel 718. Although there is work hardening, the degree is weaker, resulting in a slightly weaker yield strength and plastic-deformation-resistance ability. The stress level of Hastelloy X is even lower, with a weak yield strength and poor deformation-resistance ability during hot tension. It shows obvious plastic deformation at an earlier stage. The overall curve is relatively flat, indicating that the work-hardening effect at high temperatures is weak, and it cannot significantly increase its strength through work-hardening like Inconel 718, resulting in poor yield strength. The overall stress levels and yield strengths of FGH 4096 and FGH 4169 are both low. In the initial stage, the stress–strain relationship is approximately linear, conforming to Hooke’s law, and the materials are in the elastic-deformation stage. As the strain increases, they enter the plastic-deformation stage and undergo irreversible deformation. Under the same strain, the stress of FGH 4096 is slightly higher, with a slightly stronger yield strength and plastic-deformation-resistance ability.

It is worth noting that there are differences in the bonding strengths between these five materials and the substrate. Even if the materials themselves have not reached their yield strengths, due to insufficient bonding strength, stress concentration may occur at the interface when subjected to external forces. Once the degree of stress concentration exceeds the bearing capacity of the bonding strength, cracks will initiate, affecting the overall performance and structural stability of the materials. Therefore, the differences in mechanical behaviors reflected by the hot-tensile curves, combined with the consideration of bonding strengths, are of great significance for studying the performance of transition layer materials under actual high-temperature working conditions. This can provide references for rational material selection, optimization of the laser-cladding process, and improvement of the overall material performance, ensuring the reliability and stability of materials in practical applications.

**Figure 10:**
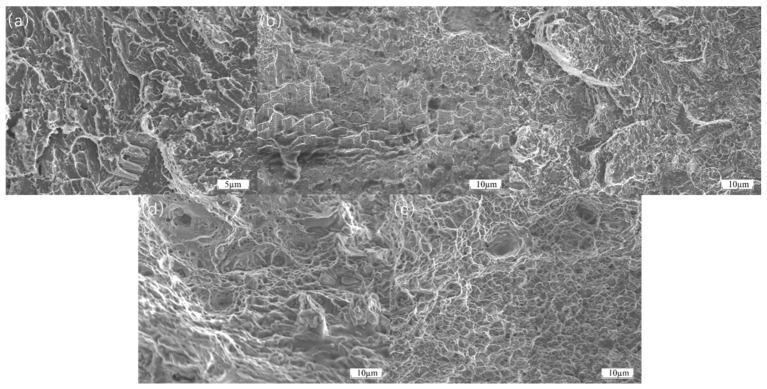
Fracture morphology of transition coatings after thermal tensile testing: (**a**) FGH4169; (**b**) FGH4096; (**c**) Hastelloy X; (**d**) Inconel 625; (**e**) Inconel 718.

Even though the thermal expansion coefficients of the different materials are not significantly different, the earliest fracture of the FGH4096 and FGH4169 transition coatings during thermal tensile testing may still be attributed to the critical factor of low interfacial bonding strength. On one hand, the inherent chemical activity of Hastelloy X and the FGH series coatings is relatively low. During laser cladding, they are less likely to undergo strong chemical reactions with the adjacent CoCrFeNiAl and the H13 substrate to form stable chemical bonds [29]. As a result, the bonding at the interface relies more on weaker physical adsorption, which is highly susceptible to failure under external forces during thermal tensile testing. Consequently, when external forces are applied and the interface is subjected to stress, cracks initiate and propagate rapidly in the interfacial regions of the FGH4096 and FGH4169 coatings due to the poor bonding strength, ultimately leading to their premature fracture. As shown in Figure 10a,b, the fracture surfaces of FGH4096 and FGH4169 exhibit complex morphological features after thermal tensile testing. In some localized areas, relatively flat fracture surfaces are observed, indicating the presence of brittle fracture components. Cracks may propagate rapidly along grain boundaries or cleavage planes, leaving insufficient time for significant plastic deformation in these regions. Simultaneously, dimple structures are observed in other areas of the fracture surfaces. These dimples are formed due to the nucleation, growth, and coalescence of microvoids during plastic deformation, suggesting that the material did not undergo complete brittle fracture but experienced some degree of plastic flow before failure. Additionally, tear ridges are visible on the fracture surfaces, resulting from the competition between plastic deformation and brittle cracking during crack propagation, reflecting the complex micromechanical behavior of the material during thermal tensile fracture.

The earliest cracking of Hastelloy X as a transition coating and the delayed cracking of Inconel 718 can be explained in terms of bonding strength and yield strength. From the initial stage of the stress–strain curve, at lower stress levels, the material undergoes elastic deformation, where stress and strain exhibit a linear relationship. During this stage, atomic bonds within the material undergo minor elastic deformation under stress, but the overall structure remains stable. As temperature increases and stress continues to rise, reaching the material’s yield strength, a distinct inflection point appears on the stress–strain curve. For the cladding materials, their yield strength during thermal tensile testing may be influenced by various factors. On one hand, if there are issues with the bonding between the cladding layer and the substrate, such as insufficient chemical bonding or the presence of microdefects, stress concentration occurs at the interface under the combined effects of thermal and tensile stresses. This stress concentration causes the local stress to far exceed the material’s macroscopic yield strength, leading to plastic deformation at lower macroscopic stress levels, manifesting as a reduction in yield strength. As stress continues to increase, the material enters the plastic deformation stage, where strain increases rapidly with stress. During this process, if the cladding layer contains inhomogeneous microstructures, such as uneven distribution of second-phase particles or the presence of pores and other defects, these areas become stress concentration points, further reducing the effective load-bearing area of the material. When the stress reaches the material’s tensile strength, defects within the material rapidly propagate, leading to crack initiation and coalescence, ultimately causing material fracture. As shown in Figure 10c–e, the fracture surfaces of Hastelloy X, Inconel 625, and Inconel 718 exhibit distinct characteristics after thermal tensile testing. The fracture surface of Hastelloy X may display relatively smooth regions due to weak interfacial bonding, alongside irregular micro-roughness resulting from some degree of plastic deformation. The fracture surface of Inconel 625 shows evident signs of plastic deformation, such as dimples of varying sizes and depths, indicating dislocation motion and material elongation before fracture. However, localized stress concentrations may also lead to the formation of fine cracks. The fracture surface of Inconel 718 is relatively uniform, with well-distributed dimples and no significant cracking at grain boundaries caused by thermal or mechanical stress concentrations, demonstrating its superior fracture resistance.

**Figure 11:**
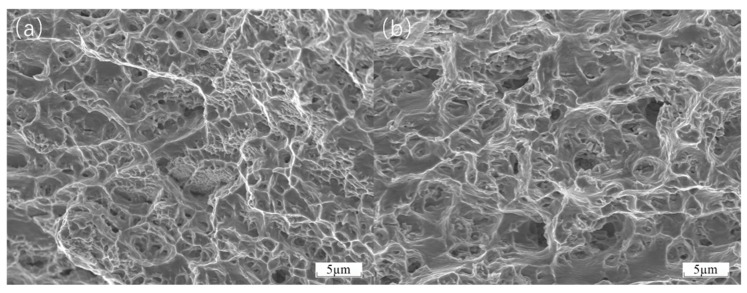
(**a**) Microstructure of the CoCrFeNiAl thermal tensile fracture surface; (**b**) fracture morphology of H13 after thermal tensile testing.

Figure 11 illustrates the microstructure of the BCC-phase CoCrFeNiAl. The image clearly reveals rich microstructural features on the material surface. Numerous dimple structures are distributed throughout the field of view, interwoven with each other, indicating that the material underwent significant plastic deformation under certain conditions. The formation of dimples is attributed to the nucleation, growth, and coalescence of microvoids within the material, which are typical microscopic indicators of ductile fracture. Additionally, tear ridges are observed interspersed among the dimples, further demonstrating localized tearing and slip phenomena during the deformation process. This image shows the ductile fracture surface of H13 after thermal tensile testing. The fracture surface exhibits typical ductile fracture characteristics, with numerous dimples of varying sizes distributed uniformly across the surface. The presence of these dimples indicates that the material underwent substantial plastic deformation before fracture. The formation of dimples is due to the nucleation, growth, and coalescence of microvoids within the material, which are key microscopic features of ductile fracture. The presence of these dimples suggests that the H13 material absorbed significant energy through plastic deformation during thermal tensile testing, delaying crack propagation and ultimately leading to ductile fracture. Furthermore, tear ridges are visible on the fracture surface, indicating that the material experienced considerable plastic deformation, with relative sliding and tearing occurring between different parts of the material. Overall, the fracture morphology fully demonstrates the excellent ductility of H13 under thermal tensile conditions, with its microstructural characteristics consistent with the mechanisms of ductile fracture.

## 4. Conclusions

This study systematically investigated five transition layer materials, namely Inconel 718, Inconel 625, Hastelloy X, FGH4096, and FGH4169, used for laser cladding between H13 steel and CoCrFeNiAl high-entropy alloy through three-point bending tests and hot tensile tests, and the following conclusions were drawn:

(1) Three-point Bending Test: The FGH4096 and FGH4169 coatings fractured first due to insufficient element diffusion and a limited number of chemical bonds at the interface, which hindered effective stress transfer. As the load increased, the interface could not endure the high tensile stress, leading to atomic bond breakage, microcrack initiation and propagation, and finally coating cracking. Their fracture surfaces exhibited a brittle-ductile mixed fracture feature, reflecting the complex mechanical behavior during the fracture process. Although Inconel 625 and Hastelloy X didn’t fracture, obvious cracks emerged at the coating-substrate interface. This was because the external load during bending generated complex stress distribution within the cladded layer. Despite similar thermal expansion coefficients reducing thermal stress, mechanical stress couldn’t be ignored. When local stress concentration reached the material’s yield strength, plastic deformation accumulated, and cracks occurred when it exceeded the fracture strength. In contrast, Inconel 718 performed remarkably well, with well-matched thermal expansion coefficients, sufficient element diffusion, good chemical bonding at the interface, and a stable overall structure, showing no fracture or cracking.

(2) Hot Tensile Test: FGH4096 and FGH4169 fractured first due to low interfacial bonding strength. Affected by factors like bonding strength and yield strength, Hastelloy X cracked first. Inconel 625, with a relatively low yield strength, underwent plastic deformation earlier during the hot-tensile process, and its fracture surface showed obvious plastic deformation signs and fine cracks. Inconel 718 had strong fracture resistance, with a regular fracture surface, uniform plastic deformation, orderly dimple distribution, and no severe cracking at the grain boundaries. The hot-tensile fracture surfaces of CoCrFeNiAl and H13 showed typical ductile-fracture characteristics, with numerous dimples and tear ridges indicating significant pre-fracture plastic deformation and good toughness.

(3) Comprehensive Evaluation: Overall, Inconel 718 demonstrated significant performance advantages in both tests and is an ideal transition layer material. Inconel 625 and Hastelloy X had issues such as insufficient element diffusion and easy crack formation, while FGH4096 and FGH4169 had low load-bearing capacity and poor stability.

This study provides a basis for the selection of laser-cladding transition layer materials and process optimization, laying a foundation for the application of laser-cladding technology. Further research can explore the long-term performance and failure mechanisms of Inconel 718 and optimize process parameters to promote the widespread application of this technology.

## Figures and Tables

**Table 1:** Composition of the substrate and the transition coating.

Alloys	Elements (wt.%)								
	Cr	Mo	Ni	Fe	C	Si	Mn	Nb	S
H13	4.75–5.50	1.10–1.75	≤1	Bal.	0.32–0.45	0.80–1.20	0.20–0.50	/	≤0.030
Inconel 718	17–21	2.8–3.3	50–55	Bal.	≤0.08	≤0.35	≤0.35	4.75–5.5	≤0.01
Inconel 625	20–23	8–10	Bal.	≤5	≤0.1	≤0.5	≤0.015	33.15–4.15	≤0.015
Hastelloy X	20.5–23	8–10	Bal.	17–20	0.05–0.15	≤1	≤1	/	≤0.03
FGH 4096	15–16.5	3.8–4.2	Bal.	≤0.5	0.02–0.05	≤0.2	≤0.15	0.6–1	≤0.015
FGH 4169	17–21	2.8–3.3	50–55	Bal.	/	≤0.35	≤0.35	4.75–5.5	≤0.015

**Table 2:** Laser-cladding process parameters.

Number	Power (W)	Scanning Speed (mm/s)	Energy Density (W∙mm^−2^)
1	1000	4	59.52
2	1000	6	39.68
3	1000	8	29.76
4	1000	10	23.81
5	1250	4	74.40
6	1250	6	49.60
7	1250	8	37.20
8	1250	10	29.76
9	1500	4	89.29
10	1500	6	59.52
11	1500	8	44.64
12	1500	10	35.71
13	1750	4	107.17
14	1750	6	69.44
15	1750	8	52.08
16	1750	10	41.67
17	2000	4	119.05
18	2000	6	79.37
19	2000	8	59.52
20	2000	10	47.62

## Data Availability

Data are contained within the article.
